# A cross-sectional survey of emergency and essential surgical care capacity among hospitals with high trauma burden in a Central African country

**DOI:** 10.1186/s12913-015-1147-y

**Published:** 2015-10-23

**Authors:** Marquise Kouo-Ngamby, Fanny Nadia Dissak-Delon, Isabelle Feldhaus, Catherine Juillard, Kent A. Stevens, Martin Ekeke-Monono

**Affiliations:** Department of Family Health, Ministry of Public Health, Yaoundé, Cameroon; Center for Global Surgical Studies, Department of Surgery, University of California, San Francisco, San Francisco, CA USA; Johns Hopkins Bloomberg School of Public Health, International Injury Research Unit, Baltimore, MD USA; Department of Surgery, Johns Hopkins Hospital, Baltimore, USA; World Health Organization, Regional Office for Africa, Brazzaville, Republic of Congo

**Keywords:** Surgery, Cameroon, Capacity, Surgical care, Infrastructure, Equipment and supplies, Human resources for health

## Abstract

**Background:**

As the overwhelming surgical burden of injury and disease steadily increases, disproportionately affecting low- and middle-income countries, adequate surgical and trauma care systems are essential. Yet, little is known about the emergency and essential surgical care (EESC) capacity of facilities in many African countries. The objective of this study was to assess the EESC capacity in different types of hospitals across Cameroon.

**Methods:**

This cross-sectional survey used the WHO Tool for Situational Analysis to Assess EESC, investigating four key areas: infrastructure, human resources, interventions, and equipment and supplies. Twelve hospitals were surveyed between August and September 2009. Facilities were conveniently sampled based on proximity to road traffic and sociodemographic composition of population served in four regions of Cameroon. To complete the survey, investigators interviewed heads of facilities, medical advisors, and nursing officers and consulted hospital records and statistics at each facility.

**Results:**

Seven district hospitals, two regional hospitals, two general hospitals, and one missionary hospital completed the survey. Infrastructure for EESC was generally inadequate with the largest gaps in availability of oxygen concentrator supply, an on-site blood bank, and pain relief management guidelines. Human resources were scarce with a combined total of six qualified surgeons, seven qualified obstetrician/gynecologists, and no anesthesiologists at district, regional, and missionary hospitals. Of 35 surgical interventions, 16 were provided by all hospitals. District hospitals reported referring patients for 22 interventions. Only nine of the 67 pieces of equipment were available at all hospitals for all patients all of the time.

**Conclusions:**

Severe shortages highlighted by this survey demonstrate the significant gaps in capacity of hospitals to deliver EESC and effectively address the increasing surgical burden of disease and injury in Cameroon. This data provides a foundation for evidence-based decision-making surrounding appropriate allocation and provision of resources for adequate EESC in the country.

## Background

The global burden of surgical disease is steadily increasing, disproportionately affecting low- and middle-income countries as many move through the epidemiological transition away from communicable towards non-communicable diseases [1]. Non-communicable diseases comprise 80 % of deaths in low- and middle-income countries, while 90 % of mortality due to unintentional injury occur in these settings [2, 3]. Trauma alone kills about 5.8 million people each year, accounting for 10 % of the world’s deaths and 32 % more than the number of fatalities that result from HIV/AIDS, malaria, and tuberculosis combined [4]. It is estimated that 11 % of the global burden of disease requires surgical treatment, predominantly those resulting from injury and malignancies [5]. Yet, the poorest third of the world’s population receive only 3.5 % of major surgical operations performed worldwide [6].

In sub-Saharan Africa, the burden of surgical disease is characterized by emergency and essential procedures requiring immediate attention, in contrast to industrialized countries where 80 % of procedures are elective [7, 8]. This is the case in Cameroon. A retrospective study of the Regional Hospital in Limbe, Cameroon found that the most frequently performed major procedures were cesarean section, circumcision, laparotomy, appendectomy, and hernia repair [7]. In 2012, injuries comprised 7.7 % of the burden of disease in the country while non-communicable diseases made up 37.5 % [9]. According to the World Health Organization, the country has an estimated mortality rate of 101.8 per 100,000 population due to injury and an estimated annual burden of disease due to injury comparable to that attributable to malaria [10, 11]. In a study creating a prospective trauma registry at the Central Hospital of Yaoundé, nearly 50 % of emergency department visits were injured patients over a time period of six months [12].

Despite this growing burden, comprehensive assessments of surgical capacity in resource-limited settings continue to report severe shortages in available resources [13–19]. Inadequacies vary in degree across areas of infrastructure, health workforce, service provision, equipment, and supplies. Reducing these gross disparities and improving access to adequate emergency and essential surgical care (EESC) is critical to alleviating the global burden of disease. Realizing universal coverage of essential surgery in low- and middle-income countries could avert an estimated 1.5 million deaths per year [20]. In Cameroon, a study assessing the compliance of district hospitals with the World Health Organization/International Association for Trauma and Intensive Care (WHO/IATSIC) “Guidelines for Essential Trauma Care” found that district hospitals surveyed continue to lack compliance, citing inadequacies in the availability and utilization of equipment and human resources training [21]. Achieving full compliance with these guidelines relating to process and outcomes relies on having adequate physical structures and human resources in place.

To date, there has not been any comprehensive assessment of surgical care capacity in terms of physical resources and service provision across different levels of the health system in Cameroon. Understanding existing EESC capacity is the foundation of evidence-based decision-making surrounding appropriate allocation and provision of resources towards greater equity in health and surgery for all. The objective of this study was to assess the EESC capacity in different types of hospitals across Cameroon.

## Methods

### Study setting

The Republic of Cameroon is a Central African country home to over 22 million people across an area of 475,650 km^2^, most densely concentrated in the Littoral and Western regions [9, 22, 23]. The median age of the population is 18.3 years, with 42.9 % of the population under the age of 15 years [9]. Urban areas contain 53 % of the population, the largest cities being the economic hub of Douala, the largest port city in the Economic and Monetary Community of Central Africa, and the political capital, Yaoundé [9]. As of 2012, with a crude birth rate of 37.7 per 1000 population, the infant mortality rate in Cameroon is 61 per 1000 live births and the under-five mortality rate is 95 per 1000 live births [24]. The life expectancy of the population at birth in 2012 was 54.6 years of age [24].

### WHO tool for situational analysis to assess emergency and essential surgical care

The WHO Tool for Situational Analysis to Assess Emergency and Essential Surgical Care, a component of the WHO Integrated Management for Emergency & Essential Surgical Care (IMEESC) toolkit, was used to assess EESC capacity in hospitals in Cameroon by investigating four areas: infrastructure, human resources, interventions, and EESC equipment and supplies [25, 26]. According to this instrument, hospitals are classified into broad categories by number of inpatient beds available (ie, 101–200, 201–300, etc.). However, the total number of hospital beds was recorded when possible. The tool queries the availability of eight types of care providers, 35 surgical interventions, and 67 items of equipment [27, 28]. Responses relating to resource availability were scored according to WHO methodology, where “0” indicates “not available,” “1” indicates “available with frequent shortages or difficulties,” and “2” indicates “fully available for all patients all the time.” Thus, higher scores represent higher availability.

### Selection of hospitals

**Fig. 1:**
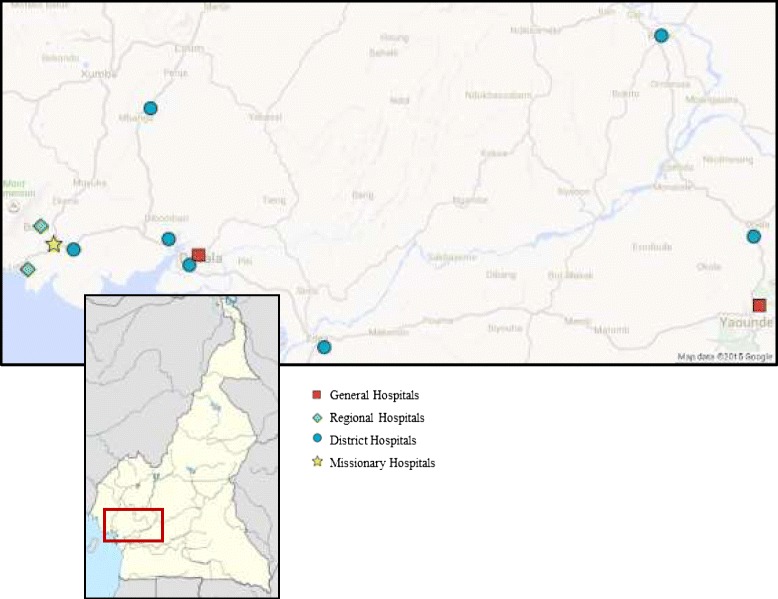
Map of Cameroon showing hospitals surveyed, August–September 2009. Map data and image sourced from Google. Usage complies with the terms and conditions specified by Google

In Cameroon, hospitals are classified according to level of care: primary level hospitals are called district hospitals; secondary level hospitals are regional hospitals; and general hospitals provide tertiary care. A total of 12 hospitals across the four most populated regions of Cameroon, with the exception of the high-risk Extreme North Region, were surveyed between August and September 2009. Hospitals were conveniently sampled based on proximity to road traffic and sociodemographic compositions of population served in four regions of Cameroon (Fig. 1). Hospitals were selected at each level of the health system based on perceived high volume of injury and trauma-related cases to assess sites that have the greatest need for EESC capacity. While a statistically representative sampling is beyond the scope of this evaluation, hospitals were selected to ensure a range of sociodemographic characteristics in the population served by these facilities. This was done to guard against undue selection bias for wealthier, urban populations that have greater access to general hospitals that typically have greater resources for emergency and essential surgical care.

In the Littoral Region, one district hospital was located on the main road between Douala and Yaoundé, another district hospital on the main road linking Douala and the West Region, and a tertiary hospital where the unsafe use of motorbikes is frequent. Two district hospitals were located in the Centre Region on the road between Yaoundé and rural areas reporting high rates of road traffic incidents. Two district, one regional, and one missionary hospital were identified near roads with high rates of traffic incidents in the Southwest Region. The two general hospitals across regions were selected based on the diversity of the population presenting to the facilities. As tertiary referral hospitals in the largest cities in the country, these two facilities receive large patient volume comprised of both initial patient presentations and district and regional referrals.

### Survey implementation

Investigators completed the survey tool during interviews of heads of facilities, medical advisors, and nursing officers, as well as through consultation of hospital records and statistics at each facility. Study investigators translated the survey tool into French. Because French is often the primary language across Cameroon, the tool was administered in French at the majority of hospital sites. Any conflicts in translation were resolved through consensus. Another investigator back translated the tool following data collection to ensure fidelity. Data on equipment declared during interviews were confirmed by observation and in discussions with heads of emergency and/or surgery units.

### Data analysis

Data were analyzed using Microsoft Excel 2008. Descriptive statistics were generated to quantitatively demonstrate the EESC capacity across hospitals in Cameroon. Frequencies were calculated to illustrate resource availability surrounding infrastructure and emergency equipment and supplies. Human resources per million population served were determined to appropriately characterize EESC capacity for respective population sizes. These densities were determined by totaling human resources by cadre and level of care per million population served, using the population served by each hospital as the denominator of the calculation. Median numbers of human resources across facilities were also calculated. Surgical interventions were categorized by type of procedure. The mean percentage of facilities performing and referring procedures were tabulated by type of hospital and overall for each group of interventions. Using the 0 to 2 scoring system of the WHO survey tool, median scores were calculated for availability of EESC equipment and supplies for resuscitation by type of hospital as well as across all hospitals surveyed. EESC equipment and supplies were categorized according to type of equipment: airways and breathing equipment, equipment for circulation, equipment for skills for management of special injuries, equipment for diagnosis and monitoring, and equipment for security of health workers. Means of these scores resulted in a composite score to describe each type of hospital and facilities overall.

This study was reviewed and received ethical approval from the Johns Hopkins Bloomberg School of Public Health Institutional Review Board, the National Ethics Committee of Cameroon, and the Ministry of Public Health, Division of Research of Cameroon.

## Results

Data were collected from all 12 hospitals; however, two district hospitals did not provide complete reports on infrastructure indicators (Table 1). Facilities most commonly failed to report on particular indicators when requested data was not regularly included in facility records. Some hospitals aggregated records and information on adult and child surgery, making differentiated analyses impossible.

**Table 1:** Profile of surveyed hospitals, 2009 (*N* = 12)

Indicator	Hospital characteristics											
Type of hospital	District	District	District	District	District	District	District	Regional	Regional	General	General	Missionary
Estimated population served	134,000	200,000^a^	150,000	200,000	56,675	500,000^a^	130,656 [30]	NR	NR	>3 mil	>3 mil	40,000
Number of beds	75	33	54	100	20–23	NR	68	200	101–200	701–1000	101–200	47
Number of admissions (per year)	501–700	NR	701–1000	1001–2000	>5000	NR	1001–2000	>5000	2001–5000	>5000	>5000	2001–5000
Number of functioning operating rooms^b^	21–50	NR	11–20	2	2	2	2	3–4	3–4	11–20	5–10	2
Number of patients requiring surgical procedures (per year)	201–300	NR	301–400	301–400	401–500	NR	21–50	501–700	701–1000	2001–5000	701–1000	1001–2000
Number of children (<15 years) requiring surgical procedures (per year)	21–50	NR	NR	21–50	81–100	NR	21–50	51–80	201–300	NR	101–200	301–400
Number of patients referred to a higher level of care for surgery (per year)	NR	NR	NR	NR	21–50	NR	21–50	NR	11–20	51–80	0	81–100
Average distance traveled to facility (km)	NR	NR	101–200	11–20	51–80	NR	2	NR	301–400	501–700	NR	21–50
Average distance traveled if referred elsewhere (km)	NR	NR	81–100	51–80	11–20	51–80	101–200	51–80	101–200	11–20	0	5–10

NR = not reported

^a^2009 population estimates using 2013/2015 data

^b^Includes major and minor operating rooms

### Profile of hospitals

Of the 12 hospitals that completed the survey, seven were district hospitals, two were regional hospitals, two were general hospitals, and one was a missionary hospital. Three hospitals are located in the Centre Region, five in the Littoral Region, and four in the Southwest Region. District hospitals estimated serving a population ranging from 56,675 to 500,000 people. Four district hospitals had over 50 beds and three reported from 501 to more than 5000 admissions annually. Four district hospitals had two functioning operating rooms, while at least two district hospitals had more than ten. District hospitals reported serving 21 to up to 500 adults (>15 years) and from 21 to 100 children (<15 years) requiring surgical procedures in the past year from August to July. Few district facilities reported data on number of patients referred to higher levels of care.

Regional hospitals reported between 2000 to more than 5000 admissions annually. The regional hospitals served the Southwest Region, totaling approximately 1.3 million inhabitants. Both regional hospitals reported having between 101 and 200 beds and 3 to 4 functioning operating rooms. They reported serving 501 to 1000 adults (>15 years) and 51 to 300 children (<15 years) requiring surgical procedures.

General hospitals estimated their population served to be greater than 3 million with more than 5000 annual admissions. One of the general hospitals reported having between 101 and 200 beds, while the other had between 701 and 1000 beds. General hospitals had between 5 and 20 functioning operating rooms and served between 701 and 5000 adults (>15 years). Among general hospitals, only one reported the number of children served at 101 to 200 children (<15 years) requiring surgical procedures. The other general hospital reported referring between 51 and 80 patients to higher levels of surgical care per year.

The single private missionary hospital surveyed reported serving a smaller population of approximately 40,000 with 21 to 50 beds. It reported admitting between 2001 and 5000 patients annually and had two functioning operating rooms. The missionary hospital served between 1001 and 2000 adults (>15 years) and 301 to 400 children (<15 years) requiring surgical procedures. Between 81 and 100 patients were referred to a higher level of care for surgery, typically between 5 and 10 km away from the hospital.

### Infrastructure

**Fig. 2:**
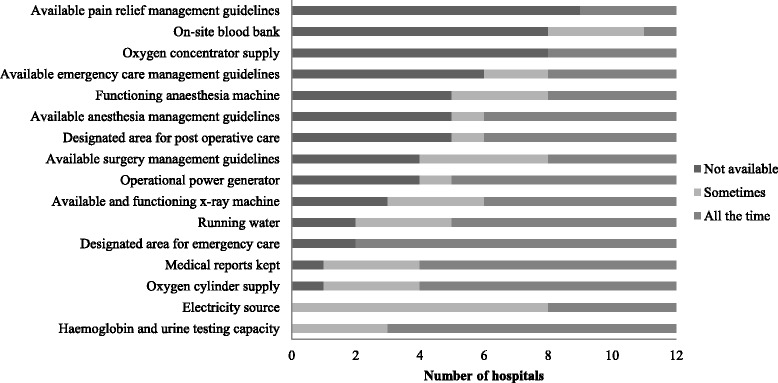
Frequency of available infrastructure for emergency and essential surgery

The infrastructure for EESC across facilities surveyed was largely inadequate with the largest gaps in the availability of oxygen concentrator supply, an on-site blood bank, and pain relief management guidelines (Fig. 2). No facility had all infrastructure recommended for EESC all of the time. All facilities had some level of access to an electricity source (33 % all the time, 67 % sometimes) and hemoglobin and urine testing capacity (75 % all the time, 25 % sometimes). Most hospitals had an available oxygen cylinder supply (67 % all the time, 25 % sometimes), designated area for emergency care (83 % all the time), and running water (58 % all the time, 25 % sometimes) as well as kept medical records (67 % all the time, 25 % sometimes).

General hospitals were most likely to have greater infrastructure for EESC with more consistency compared to other types of hospitals. The only EESC infrastructure at general facilities reported to be completely absent was an oxygen concentrator supply. All EESC infrastructure, except designated areas for emergency and postoperative care, pain relief management guidelines, and an on-site blood bank, were available at least some of the time at the missionary hospital. District hospitals were the least likely to have EESC infrastructure available with only five indicators reported to be available all of the time: oxygen cylinder supply, medical recordkeeping, designated areas for emergency and postoperative care, and hemoglobin and urine testing capacity.

### Human resources

Human resources were scarce with a combined total of six qualified surgeons, seven qualified obstetrician/gynecologists, and no anesthesiologists at district, regional, and missionary hospitals. Overall, there was a median of one qualified surgeon across hospitals. District hospitals combined reported 3.65 qualified surgeons per million population (Table 2). District hospitals also had the highest number of general doctors providing surgery and anesthesia combined at 13.86 per million population among public facilities. Across occupational categories, the general hospitals typically reported greater numbers of qualified human resources available for EESC and fewer general doctors and clinical/assistant officers providing surgical care. The general hospitals reported a median of 11.5 qualified surgeons available, the highest among the types of hospitals surveyed. Similarly, the general hospitals had a median of four qualified anesthesiologist physicians available, while other facilities reported none. The district hospitals reported 19 to 70 paramedics and/or midwives, while general hospitals reported between 271 and 300 paramedics and/or midwives available for EESC. This corresponded to 165.5 and 95.2 paramedics and/or midwives per million population for district and general hospitals, respectively.

**Table 2:** Human resources available for emergency and essential surgery per million population

Occupational categories	Type of hospital			
	District (*n* = 7)	Regional (*n* = 2)	General (*n* = 2)	Total (*N* = 11)
Qualified surgeons	3.65	0.77	3.83	8.25
Qualified anesthesiologists physicians	0.00	0.00	1.33	1.33
Qualified obstetrician/gynecologists	2.19	3.08	2.00	7.26
General doctors providing surgery	8.75	0.77	0.33	9.85
General doctors providing anesthesia	5.10	0.00	0.00	5.10
Nurse/clinical/assistant officers providing anesthesia	10.94	2.31	2.17	15.41
Clinical/assistant officers providing surgery	13.13	0.00	0.00	13.13
Paramedics/midwives	165.53	46.15^a^	95.17	306.85

^a^Only 1 regional hospital reported

Note: Missionary hospital not included in table due to skewed representation upon scaling to one million population

### Interventions

Of the 35 surgical interventions included in the survey, 16 were provided by all hospitals. Both general hospitals provided all interventions with the exception of cataract surgery, which was only available at one of the two general hospitals. The missionary hospital also provided all interventions except for urethral stricture dilatation, obstetric fistula repair, and cleft lip repair. Both regional hospitals surveyed provided 30 of the 35 interventions. Obstetric fistula repair, urethral stricture dilatation, cricothyroidotomy/tracheotomy, general anesthesia by inhalation, peripheral anesthesia blocks, and cataract surgery were least likely to be provided among district hospitals surveyed (Table 3).

**Table 3:** Overview of procedure and referral patterns: Mean percentage by type of hospital

Procedure	District (*n* = 7)		Regional (*n* = 2)		General (*n* = 2)		Missionary (*n* = 1)		Total (*N* = 12)	
	Performs	Refers	Performs	Refers	Performs	Refers	Performs	Refers	Performs	Refers
Incision and drainage of abscess	100	0	100	0	100	0	100	0	100	0
Suturing	100	0	100	0	100	0	100	0	100	0
Wound debridement	100	0	100	0	100	0	100	0	100	0
General surgery procedures^a^	100	5	100	0	100	0	100	0	100	3
Biopsy	100	14	100	0	100	0	100	0	100	8
Obstetrical/gynecology procedures^b^	82	14	100	0	100	0	75	25	88	10
Burn management^c^	93	50	100	50	100	0	100	100	96	46
Orthopedic procedures^d^	86	34	100	10	100	0	100	20	92	23
Chest tube insertion	86	29	100	0	100	0	100	0	92	17
Urology procedures^e^	79	21	100	13	100	0	75	25	85	17
Resuscitation	71	86	100	50	100	0	100	100	83	67
Pediatric surgery procedures^f^	68	39	75	25	100	0	75	50	75	31
Ear, nose, and throat procedures^g^	50	64	100	50	100	0	100	50	71	50
Anesthesia^h^	61	18	63	0	88	0	100	0	69	10
Ophthalmology procedures^i^	0	86	0	100	50	100	100	0	17	83

^a^Includes herniorrhaphy, appendectomy, laparotomy

^b^ Includes obstetric fistula repair, dilatation and curettage, cesarean section, tubal ligation

^c^ Includes acute burn management, contracture release, skin grafting

^d^Includes open and closed fracture treatment, joint dislocation, amputation, drainage of osteomyelitis or septic arthritis

^e^Includes male circumcision, hydrocele repair, cystostomy, urethral stricture repair

^f^Includes congenital hernia repair, club-foot treatment, neonatal surgery, cleft lip repair

^g^Includes removal of foreign body, cricothyroidotomy, tracheostomy

^h^Includes general, regional, spinal, and ketamine anesthesia

^i^Includes cataract removal

With respect to referrals for particular interventions, district hospitals were most likely to refer patients to other facilities for care. District hospitals reported referring patients for 22 of the 35 interventions specified by the survey. The regional and missionary hospital each reported the referral of patients for 9 of the 35 interventions. Both general hospitals only refer patients needing cataract surgery to other facilities. Among the seven district hospitals and the interventions for which they referred patients to other facilities, dilatation and curettage, cystostomy, laparotomy, joint dislocation treatment, and biopsy procedures were referred the least (Table 3). This corresponded to a greater proportion of these facilities providing these interventions.

### Emergency equipment and supplies for resuscitation

Hospitals frequently lacked equipment and supplies regardless of type of facility. No hospital had all of the emergency equipment and supplies listed for resuscitation. While no hospital had a cricothyroidotomy set, all hospitals had access to nine of the 67 pieces of equipment outlined in the survey, four of which are considered capital outlays and the remaining five being categorized as renewable items. The items least likely to be available across hospitals included Magill forceps and laryngoscope Macintosh blades, uncuffed endotracheal tubes, IV infusor bags, and chest tube insertion equipment.

District, regional, and general hospitals had the same overall median score of 1.7 associated with the availability of capital outlays, a score of 0 indicating “absent” and 2 being “fully available for all patients all of the time.” Regional hospitals received a slightly lower overall mean score of 1.4 for availability of renewable items. Scores related to availability of supplementary equipment were generally lower across hospitals than the other two equipment categories, except for regional hospitals where the mean score was also 1.4. The missionary hospital demonstrated the greatest availability of equipment and supplies for resuscitation.

Categorized by type of equipment and supplies for resuscitation (Table 4), district and missionary hospitals had the greatest availability of equipment for the security of health workers (i.e. gloves, face masks, wash basin, soap, towels, waste disposal containers, etc.) Among general hospitals, equipment for diagnosis and monitoring (i.e. stethoscope, blood pressure equipment, urinary catheter, etc.) was most often available. Airways and breathing equipment (i.e. resuscitator bag valve or mask, oxygen cylinder/concentrator, etc.) was reported to be least frequently available across all types of hospitals. The missionary hospital generally had the highest scores of availability across type of equipment and supplies for resuscitation, reporting equipment for diagnosis, monitoring, and security of health workers to always be available at the facility.

**Table 4:** Mean score of availability of essential equipment and supplies for resuscitation by type of hospital

Indicator	District (*n* = 7)	Regional (*n* = 2)	General (*n* = 2)	Missionary (*n* = 1)	Total (*N* = 12)
Airways and breathing equipment	0.88	1.19	1.40	1.73	1.13
Equipment for circulation	1.79	1.55	1.71	1.97	1.67
Equipment and skills for management of special injuries	1.66	1.45	1.61	1.90	1.58
Equipment for diagnosis and monitoring	1.82	1.32	1.77	2.00	1.61
Equipment for security of health workers	1.92	1.58	1.58	2.00	1.66
Total	1.42	1.39	1.57	1.88	1.44

Note: Indicators were scored according to the following methodology – “0” Not available; “1” Available with frequent shortages or difficulties; “2” Fully available for all patients all of the time

## Discussion

This study is the first to assess the EESC capacity with consideration of various levels of the formal health system in Cameroon. EESC capacity was lacking in all areas investigated – infrastructure, human resources, service provision, and equipment and supplies. Related studies often assess a single type of hospital in looking at EESC capacity, confining to district, regional, or general levels. Different levels of hospitals are likely to present different results because of varying levels of government resource allocation. Examining all levels of care simultaneously allows for some insight into how and the extent to which different levels interconnect, comprising the full spectrum of need and surgical care services provided within the health system. Additionally, deficiencies specific to a given level of care may be identified, which assist in more precisely targeted investments or improvements. In this way, this study was able to provide an overview in EESC capacity across different levels of the formal health system in Cameroon at facilities most likely to have the greatest burden of surgical disease and traumatic injury.

Physical infrastructure and services that were not available at lower levels of the health system were often available at higher levels of care, corresponding to historically greater government investment in these structures. While district hospitals were the least likely to have the necessary infrastructure for EESC, this corresponded with the finding that they were most likely to refer patients to higher levels of care and that general hospitals were most likely to have the essential infrastructure. Similarly, district hospitals generally had fewer qualified surgeons, anesthesiologists, and obstetricians/gynecologists corresponding to a greater number of general doctors providing surgery. This suggests that while gaps in EESC capacity exist at the district level, some level of EESC needs may still be met if patients are successfully referred and admitted to higher levels of care. These findings support formalization of referral patterns based on anticipated availability of infrastructure and services at certain levels of care; however, given the present state of the referral system and inconsistent road infrastructure, this option is not likely to address all access issues to EESC. Emphasis still needs to be put on raising the standards of the district hospitals to be capable of handling a wider range of the most prevalent surgical conditions.

Differences between EESC capacity between public and private facilities are worth noting. The private missionary hospital generally had high scores associated with availability of infrastructure, essential equipment, and supplies, and provided all surgical interventions except for three, while serving a smaller population of around 40,000. However, the hospital reported no qualified surgeons, anesthesiologists, or obstetricians/gynecologists on its staff. The ability to provide a large variety of surgical interventions despite limitations in the number of qualified human resources for surgery may be attributed to better or more efficient organization of physical and human capital. This suggests that existing staff may have particular skill sets amenable to surgery and/or that small adjustments in the process of care at district and regional hospitals can increase surgical capacity in these facilities. The increased capacity of private versus public facilities may also speak to the impact of greater revenue as a result of external sources of funding compared to public facilities.

While all four areas characterizing EESC capacity in surveyed hospitals in Cameroon were inadequate, this study identified the most significant disparity to be in availability of appropriately skilled human resources. According to a study assessing EESC capacity in Ghana, a lack of adequately skilled human resources is often the limiting factor characterizing a facility’s capacity for surgery, obstetrics, and anesthesia [29]. While overall surgical capacity is dependent on the relationship between each domain (i.e. infrastructure, human resources, interventions, equipment and supplies) weighed in the WHO survey tool, human resources play a particularly critical role because of its effect on other measures of EESC capacity captured in the WHO tool. Human resources directly affect findings surrounding interventions provided and referred at health facilities because the measure relies on whether providers have the skills to perform these services. Infrastructure, equipment, and supplies remain inconsequential to EESC delivery without skilled human resources providing relevant services appropriately. Focused efforts and investment in surgical education and training as well as the adoption of workforce policies incentivizing employment in high-burden geographical areas may reduce disparities.

Qualified human resources for EESC were extremely scarce given the large population served. Still, with the human resources that currently exist, solutions include appropriate attention and investment in adequate infrastructure, equipment, and supplies. In a study of Cameroon’s district hospitals in its Centre Region, investigators note that gaps in equipment, supplies, and service delivery could be addressed. Though specific specialty training is generally scarce in low-income settings, existing staff appear to have appropriate training in the use of essential equipment that is, if infrequently, available [21]. More efficient organization of health workforce rosters that aims at ensuring permanent presence of existing facility staff is also cited as a possible step toward narrowing these gaps [21]. Addressing shortages will allow for corresponding increases in compliance with WHO/IATSIC guidelines toward more appropriate surgical care in this setting.

Studies in other sub-Saharan African countries using the WHO Tool for Situational Analysis to Assess EESC also report generally poor levels of EESC capacity, highlighting disparities at varying degrees and at different levels of the formal health system. The tool has been determined highly reliable in measures of structure and setting, such as physical and human capital [26]. In Tanzania, a study determined that significant gaps existed for EESC capacity in first-referral health facilities, most prominently in essential equipment, infrastructure, and human resources [27]. Use of the WHO tool in Somalia presented inadequacies in essential equipment, service provision, and infrastructure [28]. A study in the Gambia found critical deficits in all of the four areas [17].

A limitation associated with this cross-sectional survey is that it provides only a snapshot in time of the surgical care capacity across Cameroon. Nevertheless, it highlights the significant gap between population needs and hospital resources, identifying where strategic efforts for capacity building and systems strengthening should focus for impact. Of the 183 public hospitals around the country, the number included in this survey is relatively few; however, because of the convenience sampling design, those selected are considered to be those with the greatest burden of emergency and trauma-related injury cases. As a result, an initial assessment of the EESC capacity of these particular facilities is critical. Future research opportunities exist to assess EESC capacity at a greater number of facilities. Though producing high reliability regarding physical capital, the WHO survey tool used in this study does not robustly examine processes and outcomes of care [26]. Additional examination into processes of care may account for discrepancies in findings between different types of hospitals in this study. Thus, this study takes into account only the physical infrastructure, human resources, and equipment and supplies reported at each surveyed facility to characterize EESC capacity. A number of hospitals were also not able to report all indicators because the requested data was not available at the time of interview or not regularly recorded in facility records. Future assessments of EESC may benefit from increased emphasis on the implementation of standardized recordkeeping instruments, such as by the utilization of a national-level surgical care registry.

## Conclusions

Severe disparities in infrastructure, human resources, service provision, and essential equipment and supplies demonstrate the significant gaps in capacity of hospitals to deliver EESC and effectively address the increasing surgical burden of disease and injury in Cameroon. As the foundation for appropriate resource allocation and service provision for EESC, this study emphasizes the continued need for investment in EESC infrastructure, equipment and supplies, and appropriately qualified surgeons, anesthesiologists, and obstetricians/gynecologists.

Since data collection, there has been notable political will and stakeholder interest in improving the EESC capacity of health facilities around the country. This baseline data serves as a starting point from which to make informed decisions. As the surgical burden of disease continues to increase, there is a continued need for comprehensive, rigorous assessment of EESC capacity in Cameroon as a mechanism highlighting where resources are needed and where investment will prove most valuable. Future longitudinal follow-up assessments will also highlight which interventions or investments result in the greatest improvement in Cameroon’s hospital-based resources for the delivery of surgical care.

## Abbreviations

EESC: Emergency and essential surgical care
IATSIC: International Association for Trauma and Intensive Care
IMEESC: Integrated Management for Emergency & Essential Surgical Care
WHO: World Health Organization

## References

[1] Murray CJL, Vos T, Lozano R, Naghavi M, Flaxman AD, Michaud C, Ezzati M, Shibuya K, Salomon JA, Abdalla S, Aboyans V, Abraham J, Ackerman I, Aggarwal R, Ahn SY, Ali MK, Alvarado M, Anderson HR, Anderson LM, Andrews KG, Atkinson C, Baddour LM, Bahalim AN, Barker-Collo S, Barrero LH, Bartels DH, Basáñez M-G, Baxter A, Bell ML, Benjamin EJ (2012). Disability-adjusted life years (DALYs) for 291 diseases and injuries in 21 regions, 1990–2010: a systematic analysis for the Global Burden of Disease Study 2010. Lancet.

[2] Daar AS, Singer PA, Persad DL, Pramming SK, Matthews DR, Beaglehole R, Bernstein A, Borysiewicz LK, Colagiuri S, Ganguly N, Glass RI, Finegood DT, Koplan J, Nabel EG, Sarna G, Sarrafzadegan N, Smith R, Yach D, Bell J (2007). Grand challenges in chronic non-communicable diseases. Nature.

[3] Nantulya VM, Reich MR (2002). The neglected epidemic: road traffic injuries in developing countries. BMJ.

[4] WHO | Injuries and violence: the facts. World Health Organization; 2015. [http://www.who.int/violence_injury_prevention/key_facts/en/]

[5] Debas HT, Gosselin R, McCord C, Thind A, Jamison DT, Breman JG, Measham AR, Alleyne G, Claeson M, Evans DB, Jha P, Mills A, Musgrove P (2006). Surgery. Disease Control Priorities in Developing Countries.

[6] Weiser TG, Regenbogen SE, Thompson KD, Haynes AB, Lipsitz SR, Berry WR, Gawande AA (2008). An estimation of the global volume of surgery: a modelling strategy based on available data. Lancet.

[7] Chichom Mefire A, Atashili J, Mbuagbaw J (2013). Pattern of surgical practice in a regional hospital in Cameroon and implications for training. World J Surg.

[8] Humber N, Frecker T (2008). Rural surgery in British Columbia: Is there anybody out there?. Can J Surg.

[9] Global Health Observatory Data Repository. Geneva: World Health Organization; 2015 .

[10] The World Health Report 2004 - Changing History. World Health Organization; 2004. [http://www.who.int/whr/2004/annex/en/]

[11] WHO | Mortality and global health estimates. World Health Organization; 2015. [http://www.who.int/gho/mortality_burden_disease/en/]

[12] Juillard CJ, Stevens KA, Monono ME, Mballa GAE, Ngamby MK, McGreevy J, Cryer G, Hyder AA (2014). Analysis of prospective trauma registry data in Francophone Africa: a pilot study from Cameroon. World J Surg.

[13] LeBrun DG, Chackungal S, Chao TE, Knowlton LM, Linden AF, Notrica MR, Solis CV, McQueen KAK (2014). Prioritizing essential surgery and safe anesthesia for the Post-2015 Development Agenda: operative capacities of 78 district hospitals in 7 low- and middle-income countries. Surgery.

[14] Henry JA, Frenkel E, Borgstein E, Mkandawire N, Goddia C. Surgical and anaesthetic capacity of hospitals in Malawi: key insights. Health Pol Plan. 2015; 30:985-994. 10.1093/heapol/czu102 PMC4559113 25261799

[15] Knowlton LM, Chackungal S, Dahn B, LeBrun D, Nickerson J, McQueen K (2013). Liberian surgical and anesthesia infrastructure: a survey of county hospitals. World J Surg.

[16] Petroze RT, Nzayisenga A, Rusanganwa V, Ntakiyiruta G, Calland JF (2012). Comprehensive national analysis of emergency and essential surgical capacity in Rwanda. Br J Surg.

[17] Iddriss A, Shivute N, Bickler S, Cole-Ceesay R, Jargo B, Abdullah F, Cherian M (2011). Emergency, anaesthetic and essential surgical capacity in the Gambia. Bull World Health Organ.

[18] Kushner AL, Cherian MN, Noel L, Spiegel DA, Groth S, Etienne C (2010). Addressing the Millennium Development Goals from a surgical perspective: essential surgery and anesthesia in 8 low- and middle-income countries. Arch Surg Chic Ill 1960.

[19] Kingham TP, Kamara TB, Cherian MN, Gosselin RA, Simkins M, Meissner C, Foray-Rahall L, Daoh KS, Kabia SA, Kushner AL (2009). Quantifying surgical capacity in Sierra Leone: a guide for improving surgical care. Arch Surg Chic Ill 1960.

[20] Mock CN, Donkor P, Gawande A, Jamison DT, Kruk ME, Debas HT. Essential surgery: key messages from Disease Control Priorities, 3rd edition. The Lancet. 2015; 385:2209-2219. 10.1016/S0140-6736(15)60091-5 PMC7004823 25662414

[21] Chichom-Mefire A, Mbarga-Essim NT, Monono ME, Ngowe MN (2014). Compliance of district hospitals in the Center Region of Cameroon with WHO/IATSIC guidelines for the care of the injured: a cross-sectional analysis. World J Surg.

[22] Du Cameroun R (2012). Enquête Démographique et de Santé et À Indicateurs Multiples (EDS-MICS) 2011.

[23] Annuaire Statistique Du Cameroun 2013. Yaoundé, Cameroon: Institut National de la Statistique; 2014.

[24] Cameroon*:* Statistics. UNICEF; 2013. [http://www.unicef.org/infobycountry/cameroon_statistics.html]

[25] WHO | Integrated Management for Emergency and Essential Surgical Care (IMEESC) toolkit. World Health Organization; 2015. [http://www.who.int/surgery/publications/imeesc/en/]

[26] Osen H, Chang D, Choo S, Perry H, Hesse A, Abantanga F, McCord C, Chrouser K, Abdullah F (2011). Validation of the World Health Organization tool for situational analysis to assess emergency and essential surgical care at district hospitals in Ghana. World J Surg.

[27] Penoyar T, Cohen H, Kibatala P, Magoda A, Saguti G, Noel L, Groth S, Mwakyusa DH, Cherian M (2012). Emergency and surgery services of primary hospitals in the United Republic of Tanzania. BMJ Open.

[28] Elkheir N, Sharma A, Cherian M, Saleh OA, Everard M, Popal GR, Ibrahim AA (2014). A cross-sectional survey of essential surgical capacity in Somalia. BMJ Open.

[29] Choo S, Perry H, Hesse AAJ, Abantanga F, Sory E, Osen H, Fleischer-Djoleto C, Moresky R, McCord CW, Cherian M, Abdullah F (2010). Assessment of capacity for surgery, obstetrics and anaesthesia in 17 Ghanaian hospitals using a WHO assessment tool. Trop Med Int Health TM IH.

[30] Folefack Tengomo GL (2009). Évaluation de La Couverture Vaccinale Chez Les Enfants de 12 À 23 Mois et Les Raisons de Non Vaccination Dans Le District de Santé de TIKO (Cameroun) En 2008.

